# Supplementary figures and images for: Efficacy and safety of creatine phosphate sodium in the treatment of viral myocarditis: A systematic review and meta-analysis
Source: PLoS One. 2025 Jan 24;20(1):e0317498. doi: 10.1371/journal.pone.0317498 (PMC11760627; doi:10.1371/journal.pone.0317498)

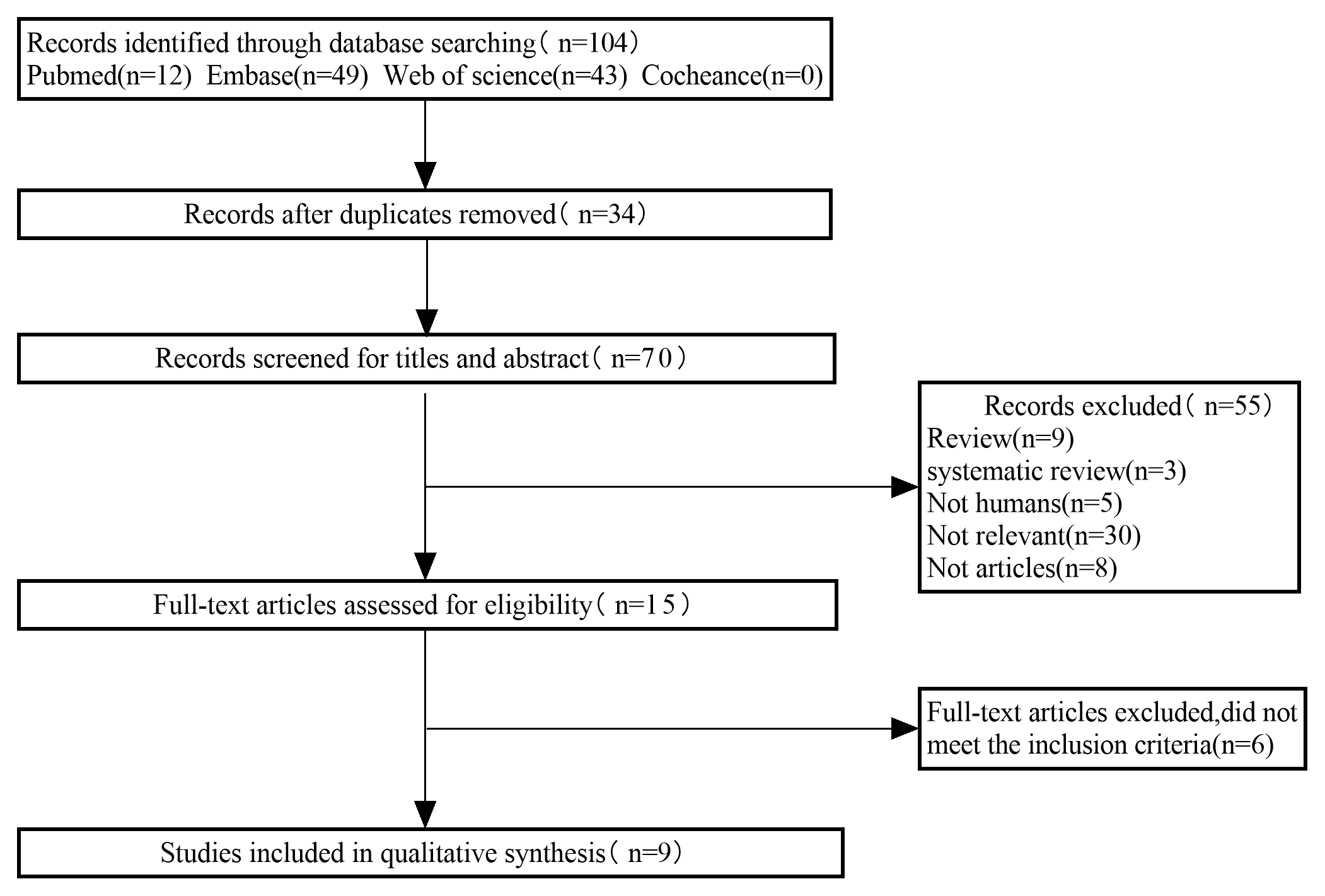

Supplement: S1 Fig — (TIF) [file pone.0317498.s004.tif]

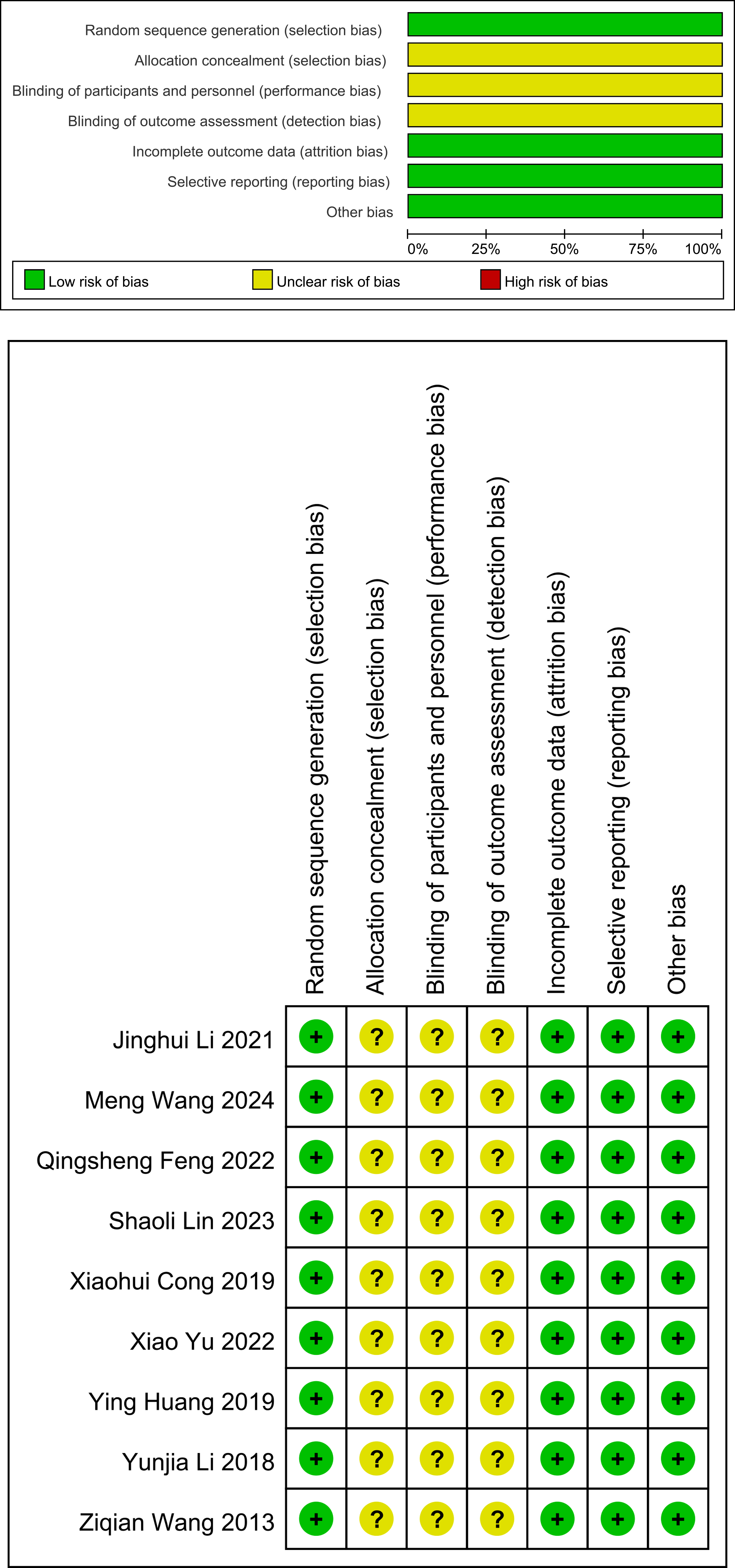

Supplement: S2 Fig — (a). The judgment of each bias risk item is expressed in percentage in all included studies.(b). Risk of bias summary. (TIF) [file pone.0317498.s005.tif]
